# Prenatal ultrasound findings in Koolen‐de Vries foetuses: Central nervous system anomalies are frequent markers of this syndrome

**DOI:** 10.1002/mgg3.1649

**Published:** 2021-03-18

**Authors:** Fe Amalia García‐Santiago, Cristina Martínez‐Payo, Elena Mansilla, Fernando Santos‐Simarro, Miguel Ruiz de Azua Ballesteros, María Ángeles Mori, Eugenia Antolín Alvarado, Yolanda Nieto, Isabel Vallcorba, Jair Tenorio, Julián Nevado, Pablo Lapunzina

**Affiliations:** ^1^ INGEMM Institute of Medical and Molecular Genetics‐IdiPAZ Hospital Universitario La Paz Madrid Spain; ^2^ Universidad Autónoma de Madrid Madrid Spain; ^3^ Centro de Investigación Biomédica en Red de Enfermedades Raras (CIBERER) Unidad 753, ISCIII Madrid Spain; ^4^ Department of Gynecology and Obstetrics Hospital Universitario Puerta de Hierro Madrid Spain; ^5^ Department of Gynecology and Obstetrics Hospital Universitario La Paz Madrid Spain; ^6^ The European Reference Network on Intellectual Disability, TeleHealth and Congenital Anomalies (ERN ITHACA) Brussels Belgium

**Keywords:** 17q21.31 microdeletion, genomic imbalance, Koolen‐de Vries syndrome, prenatal diagnosis, prenatal ultrasound, ventriculomegaly

## Abstract

**Objective:**

Prenatal diagnoses of microdeletion syndromes without ultrasound findings in the first and second trimester are always difficult. The objective of this study is to report the prenatal ultrasound findings in four foetuses diagnosed with 17q21.31 microdeletions (Koolen‐de Vries syndrome) using chromosomal microarrays (CMA).

**Patients and Methods:**

We present four foetuses with 17q21.31 microdeletion. All showed CNS anomalies in the third trimester, three had ventriculomegaly, and one hypogenesis of corpus callosum at 31 weeks of pregnancy.

**Results:**

Array‐SNPs and CGH‐array were performed on uncultured amniocytes and peripheral blood revealing a 17q21.31 microdeletion.

**Conclusions:**

Prenatal CNS anomalies (mainly ventriculomegaly) at third trimester, in spite of isolate, should be considered a prenatal ultrasound marker of this syndrome. This kind of malformations raise the possibility of an underlying genetic conditions including 17q21.31 microdeletion; thus, CMA should be taken into consideration when offering prenatal genetic counselling.

## Graphical abstract

Prenatal CNS anomalies (mainly ventriculomegaly) at third trimester, should be considered a prenatal ultrasound marker of this syndrome. This kind of malformations raise the possibility of an underlying genetic condition including 17q21.31 microdeletion. Thus CMA should be taken into consideration when offering prenatal genetic counselling.

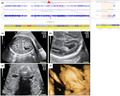

## 1 INTRODUCTION

Koolen‐de Vries syndrome is a genomic disorder characterized by hypotonia, distinctive facial features, including tall, broad forehead, long face, upslating palpebral fissures, epicanthal folds, tubular nose with bulbous nasal tip, large ears and moderate to severe intellectual disability (ID). More variable features include cardiac or genitourinary anomalies and seizures (Koolen et al., 2006, 2008).

It is usually recognized postnatally in children with the typical clinical findings and/or in individuals with ID tested by means of chromosomal microarrays (CMA; arrayCGH or SNParray). However, there are very few cases diagnosed prenatally since the changes in the prenatal age are very subtle and most of the time they are not observable until the third trimester. The decision to perform an invasive prenatal diagnosis test after the observation of these subtle ultrasound abnormalities found in the third trimester of pregnancy often leads gynaecologists and parents to indecision, and sometimes either physicians or the couple choose wait until the birth of the baby.

We here report on four patients with Koolen‐de Vries syndrome diagnosed with CMA. Three of them showed ventriculomegaly in the third trimester of pregnancy, and one hypogenesis of corpus callosum and ventriculomegaly was established in the newborn at 31 weeks of gestation.

## 2 PATIENTS AND METHODS

### 2.1 Clinical descriptions

All patients were referred for genetic assessment, one of them by a gynaecologist and the rest by neonatologists at birth. All patients were born to healthy, non‐consanguineous parents without significant family history unless noted otherwise (Table 1).

**TABLE 1:** Clinical findings in foetus with Koolen‐de Vries Syndrome. IUGR: intrauterine growth restriction. ASD: Atrial Septal Defect

	1st trimester		2st trim.	3st trimester				Birth
	T.N.	Risk		Brain	Kidney and urologic anomalies	Heart defects	IUGR	
Foetus 1 arr 17q21.31(43717703–44210822)x1	1.4 mm	+21 1/7304 +18 1/2258	—	Unilateral mild ventriculomegaly	bilateral hidronephrosis grade II	No	—	‐‐
Foetus 2 arr 17q21.31(43685925–44194835)x1	2.06 mm	+21 1/6975 +18 < 1/10000		Bilateral moderate ventriculomegaly	Hepatomegaly	No	—	2.888 gr (10 th), 48 cm (10th), PC: 35.5 cm (75–90 th) ASD, Mild bilateral ventriculomegaly
Foetus 3 arr 17q21.31(43698122–44199345)x1	2.2 mm	+21 1/458 +18 < 1/10000	—	Bilateral mild ventriculomegaly	—	—	—	2.880 gr (10th)
Foetus 4 arr 17q21.31(43706886–44210822)x1	4.2 mm	No available hiperechogenic focus on left ventricle	—	—	—	—	Severe	1.120 gr ASD, supravalvar estenosis Ureterovesical reflux Dysgenesis of corpus callosum Bilateral hippocampal dysplasia

### 2.2 Patient 1

A 33‐year‐old woman at 26 + 5 weeks of gestation was referred to our centre because of the ultrasound examination revealed a bilateral, grade II hydronephrosis and unilateral mild ventriculomegaly, which was estimated at 10.2 mm. Her husband was 37 years old, and the couple was unrelated. The family history was unremarkable, and no teratogenic exposure was noted. At 12 + 4 weeks of gestation, nuchal translucency was 1.4 mm (50th centile), and crown‐rump length was 61 mm. Based on these measurements, hormones and maternal age, the risk for trisomy 21 was 1:7,304 and for trisomy 18 as 1:2,258. A CMA in amniotic fluid reported the diagnosis of Koolen‐De Vries syndrome.

### 2.3 Patient 2

This female newborn was referred to us with hypotonia and an abnormal phenotype. She was the second child of an unrelated the couple; her mother was 32 years old. The family history was unremarkable, and no teratogenic exposure was noted. At 12 + 2 weeks of gestation, nuchal translucency was 2.06 mm (p70) and crown‐rump length was 64 mm. The risk for trisomy 21 was 1/6,975 and for trisomy 18 < 1/10,000. Ultrasound examination at 32 + 4 weeks revealed a bilateral ventriculomegaly, right ventricle 12.5 mm and left ventricle 12.8 mm. At 35 + 3 weeks, hepatomegaly and placental calcifications were also detected; thus, an infection‐work up was requested, eventually ruling out infectious diseases. Weight at birth (40 + 4 weeks) was 2,888 gr (10–25th centile), length 48 cm (10th centile), and OFC 35.5 cm (75–90th centile). Echocardiography revealed an atrial septal defect. Mild bilateral ventriculomegaly was observed at birth, (right 31 mm and left 28.7 mm). A CMA in blood at birth confirmed the diagnosis of Koolen‐De Vries syndrome.

### 2.4 Patient 3

This boy was born at 38 weeks of gestation showing hypotonia and a peculiar phenotype. Weight at birth was 2,880 gr. (10th centile). The couple was unrelated. The family history was unremarkable, and no teratogenic exposure was noted. The nuchal translucency was 2.2 mm at the first trimester. The risk for trisomy 21 was 1/458 and for trisomy 18 < 1/10,000. Non‐Invasive Prenatal Test was performed for chromosomes 13, 18 and 21, informing low risk for abnormalities of these chromosomes. Ultrasound examination revealed a bilateral ventriculomegaly at 22 weeks, right and left ventricle 12 mm. A CMA in blood after birth confirmed the diagnosis of Koolen‐De Vries syndrome.

### 2.5 Patient 4

This preterm newborn was born at 31 weeks of gestation due to severe intrauterine growth retardation, oligoamnios and risk of loss of foetal well‐being. Birthweight was 1,120 gr. and length of 40 cm. A nuchal translucency of 4.2 mm and a hyperechogenic focus on left ventricle had been reported at 12 weeks of gestation. In the neonatal period the baby had hypotonia, a mild supravalvar pulmonary stenosis plus atrial septal defect, ostium secundum type, unilateral grade II‐III ureterovesical reflux, severe dysgenesis of corpus callosum and bilateral hippocampal dysplasia. A CMA in peripheral blood confirmed the diagnosis of Koolen‐De Vries syndrome.

### 2.6 Methods

A genome‐wide scan of 850,00 tag SNPs was conducted on three patients, using the Illumina Infinium CytoSNP‐850 k BeadChip according to the manufacturer's specifications (Illumina). GenCall scores <0.15 at any locus were considered ‘no calls. Image data were analysed using the Chromosome Viewer tool contained in Genome Studio (Illumina). In addition, an allele frequency analysis was applied for all SNPs. All genomic positions were based upon NCBI Build 37 (dbSNP version 130). In patient number 3 used array‐CGH KaryoNim Prenatal 60 K was performed.

## 3 RESULTS

**FIGURE 1:**
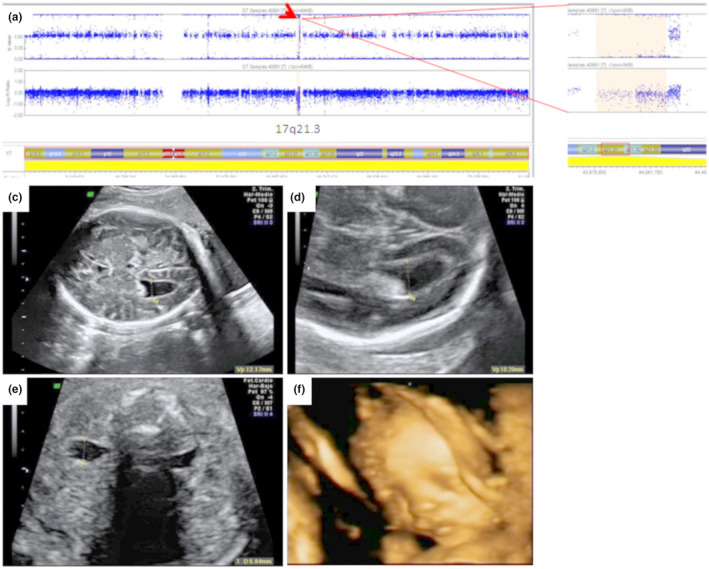
Fig. 1: A: Dark area indicates the genomic rearrangement in deletion at 17q21.31. The metric used was the log R ratio which is the log (base 2) ratio of the observed normalized R value for a SNP divided by the expected normalized R value (under manufacturer’s specifications). B: moderate bilateral ventriculomegaly in foetus 2. C: mild unilateral ventriculomegaly in foetus 1. D: bilateral hydronephrosis grade II in foetus 1. E: 3D US in surface rendering mode shows mild hypertelorism and broad nasal bridge in foetus 1.

The CMA data confirmed the presence of a 17q21.31 deletion in all our patients. The deletions extended from 41.07 to 41.57 Mb, within band 17q21.31. Figure 1 indicates the sizes and positions of the deletions. These deletions were confirmed by FISH using BAC clone MAPT (Agillent SureFISH, USA) located within the deleted region. The parents’ karyotypes were all normal, and FISH analyses using the same clone were also normal.

## 4 DISCUSSION

ID occurs in approximately 2%–3% of the general population (Leonard & Wen, 2002). Almost any cases show ultrasound alterations or these alterations do not appear until the third trimester. Genomic imbalances are a major cause of congenital and developmental abnormalities observed in patients with dysmorphic features, ID, autistic spectrum disorders (ASDs) and/or multiple congenital anomalies (MCAs). Array comparative genomic hybridization (aCGH) is a powerful molecular tool to detect and study genomic imbalances, disease mechanisms and pathogenesis; which is rapidly becoming as a new gold standard in postnatal cases (Miller et al., 2010; Vallespin et al., 2013) and in prenatal cases with congenital malformations (Armengol et al., 2012). We here correlated specific prenatal ultrasound findings (CNS) with a microdeletion syndrome (17q21.31 deletion; Koolen de Vries syndrome) and consequently to help parents in the prenatal counselling.

The 17q21.31 microdeletion syndrome (Koolen‐de Vries syndrome) is a clinically recognizable genomic disorder (MIM 610443) (Koolen et al., 2006; Sharp et al., 2006). The common phenotype observed in almost all affected individuals includes developmental delay, hypotonia, a friendly/amiable behaviour and mild and characteristic dysmorphic facial features (Koolen et al., 2008, 2016). These authors included other features as ventriculomegaly (38%), skeletal abnormalities (scoliosis/kyphosis [36%], cryptorchidism [78%], kidney/urologic defects [32%]). The minimal critical region that is recurrently deleted in individuals with the 17q21.31 microdeletion syndrome is a 478‐kb region, which is thought to result from non‐allelic homologous recombination, mediated by flanking low‐copy repeats (Koolen et al., 2006). This region encompasses six genes, including *C17orf69*, the corticotrophin releasing hormone receptor 1 gene (*CRHR1*) (MIM 122561) and the microtubule associated protein tau gene (*MAPT*) (MIM 157140) (Koolen et al., 2008). These authors established that the haploinsufficiency of one or more of these genes might underlie the phenotype seen in the 17q21.31 syndrome. In addition, Tan et al. (2009), Zollino et al. (2012) and Koolen et al. (2012) included *KANSL1* gen in this critical region of this syndrome. The *KANSL1* gene encodes a nuclear protein that plays a role in chromatin modification. It is a member of a histone acetyltransferase (HAT) complex (Smith et al., 2005), detected high expression in liver and intermediate expression in all other adult and foetal brain. All these authors evidencing finally that the patients which had mutations within *KANSL1* had phenotypic traits of this syndrome.

Review of previously reported cases of 17q21.31 microdeletion syndrome (Egloff et al., 2014) revealed that although the diagnosis was made postnatally in most, a significant proportion (33.8%) had prenatal brain findings, suggesting that the diagnosis could have been made at an earlier stage (Dornelles‐Wawruk et al., 2013; Dubourg et al., 2011; Egloff et al., 2014; El CFhehadeh‐Djebbar et al., 2011; Jaillard et al., 2010; Kitsiou‐Tzeli et al., 2012; Koolen et al., 2008, 2012; Sharkey et al., 2009; Shaw‐Smith et al., 2006; Tan et al., 2009; Terrone et al., 2012; Wray, 2013; Wright et al., 2011).

Enlargement of the ventricles may occur for a number of reasons, such as loss of brain volume (perhaps due to infection or infarction), or impaired outflow or absorption of cerebrospinal fluid from the ventricles. Often, however, there is no identifiable cause. This diagnosis is generally found in the routine foetal anomaly scan at 18–22 weeks of gestation. It is one of the most common abnormal brain findings on prenatal ultrasound, occurring in around 1–2 per 1,000 pregnancies (Achiron et al., 1993; Tomlinson et al., 1997). In many cases of mild ventriculomegaly, however, there is resolution during the pregnancy. In patient 4 although no ventriculomegaly was described in prenatal US, a diagnosis of dysgenesis of corpus callosum was established in the neonatal period. Septal heart defects were found in two cases at birth (atrial septal defects). These kinds of heart defects are very difficult to detect in prenatal ultrasound because the foramen oval is always open in foetal life. One of the foetuses showed bilateral hydronephrosis grade II. This pregnancy was interrupted; for this reason, we did not have data at birth of this patient.

The observation of minor malformations in prenatal diagnosis, especially in pregnancies over 22 weeks, is a source of uncertainly when it comes to genetic analysis. However, these findings are the only prenatal expression of many genetic disorders, as many of them do not present major malformations in this period. From our point of view, it is important to evaluate each case individually when counselling parents and to inform them about the possibility of invasive genetic tests in order to rule out microdeletions and microduplication syndromes. This is especially relevant for those syndromes with severe consequences in postnatal life.

In summary, it has been shown that CNV occur in about 0.4% of pregnancies and are unrelated to maternal age or scape to classical aneuploidy screening. We reported the observation of CNS abnormalities in prenatal diagnosis of foetuses with 17q21.31 deletion syndrome. Combined with imaging examination, the application of CMA improves the diagnosis of submicroscopic chromosomal aberrations in foetuses with congenital anomalies. Thus, prenatally detected CNS (mainly ventriculomegaly and abnormalities of corpus callosum) should orientate gynaecologists and obstetricians to the possibility of a microdeletion/microduplication syndrome and consequently to offer invasive diagnostic methods to the couple. Prenatal ventriculomegaly should be added as a marker of the Koolen‐de Vries syndrome.

## 5 STATEMENT OF ETHICS

The research was conducted ethically in accordance with the World Medical Association Declaration of Helsinki. The paper is exempt from ethical committee approval, because the manner in which it proceeds is part of the usual clinical practice. Parents have signed express consent for the case to be published.

## CONFLICT OF INTEREST

The authors declare no conflict of interest to declare.

## AUTHOR CONTRIBUTIONS

Fe Amalia García‐Santiago: conception, design of the work, the acquisition and analysis, and interpretation of data. Cristina Martínez‐Payo: analysis and interpretation of ultrasound examinations. Elena Mansilla Aparicio: revising it critically for important intellectual content. Fernando Santos‐Simarro: newborn screening. Miguel Ruiz de Azua Ballesteros: analysis and interpretation of ultrasound examinations. María de los Angeles Mori Álvarez: analysis and interpretation of data. Eugenia Antolín Alvarado: revising it critically for important intellectual content. Yolanda Nieto: analysis and interpretation of ultrasound examinations. Isabel Vallcorba: analysis and interpretation of data. Jair Tenorio: figure designe. Julian Nevado: analysis and interpretation of data. Pablo Lapunzina: revising it critically for important intellectual content.

## Data Availability

Data are openly available in a public repository that issues data sets with DOIs.

## References

[mgg31649-bib-0001] Achiron, R. , Schimmel, M. , Achiron, A. , & Mashiach, S. (1993). Fetal mild idiopathic lateral ventriculomegaly: is there a correlation with fetal trisomy? Ultrasound in Obstetrics and Gynecology, 3(2), 89–92. 12797298 10.1046/j.1469-0705.1993.03020089.x

[mgg31649-bib-0002] Armengol, L. , Nevado, J. , Serra‐Juhe, C. , Plaja, A. , Mediano, C. , Garcia‐Santiago, F. A. , Garcia‐Aragones, M. , Villa, O. , Mansilla, E. , Preciado, C. , Fernandez, L. , Angeles Mori, M. , Garcia‐Perez, L. , Lapunzina, P. D. , & Perez‐Jurado, L. A. (2012). Clinical utility of chromosomal microarray analysis in invasive prenatal diagnosis. Human Genetics, 131(3), 513–523. 21975797 10.1007/s00439-011-1095-5 PMC3277707

[mgg31649-bib-0003] Dornelles‐Wawruk, H. , Pic‐Taylor, A. , Rosenberg, C. , Krepischi, A. C. , Safatle, H. P. , Ferrari, I. , & Mazzeu, J. F. (2013). Complex phenotype associated with 17q21.31 microdeletion. Molecular Syndromology, 4(6), 297–301. 24167466 10.1159/000354120 PMC3776469

[mgg31649-bib-0004] Dubourg, C. , Sanlaville, D. , Doco‐Fenzy, M. , Le Caignec, C. , Missirian, C. , Jaillard, S. , Schluth‐Bolard, C. , Landais, E. , Boute, O. , Philip, N. , Toutain, A. , David, A. , Edery, P. , Moncla, A. , Martin‐Coignard, D. , Vincent‐Delorme, C. , Mortemousque, I. , Duban‐Bedu, B. , Drunat, S. , … Andrieux, J. (2011). Clinical and molecular characterization of 17q21.31 microdeletion syndrome in 14 French patients with mental retardation. European Journal of Medical Genetics, 54(2), 144–151. 21094706 10.1016/j.ejmg.2010.11.003

[mgg31649-bib-0005] Egloff, M. , Encha‐Razavi, F. , Garel, C. , Bonniere‐Darcy, M. , Millischer, A. E. , Lapierre, J. M. , Fontaine, S. , de Blois, M. C. , Vekemans, M. , Turleau, C. , Ville, Y. , & Malan, V. (2014). 17q21.31 microdeletion: brain anomalies leading to prenatal diagnosis. Cytogenetic and Genome Research, 144(3), 178–182. 25402493 10.1159/000369117

[mgg31649-bib-0006] El Chehadeh‐Djebbar, S. , Callier, P. , Masurel‐Paulet, A. , Bensignor, C. , Mejean, N. , Payet, M. , Ragon, C. , Durand, C. , Marle, N. , Mosca‐Boidron, A. L. , Huet, F. , Mugneret, F. , Faivre, L. , & Thauvin‐Robinet, C. (2011). 17q21.31 microdeletion in a patient with pituitary stalk interruption syndrome. European Journal of Medical Genetics, 54(3), 369–373. 21397059 10.1016/j.ejmg.2011.03.001

[mgg31649-bib-0007] Jaillard, S. , Drunat, S. , Bendavid, C. , Aboura, A. , Etcheverry, A. , Journel, H. , Delahaye, A. , Pasquier, L. , Bonneau, D. , Toutain, A. , Burglen, L. , Guichet, A. , Pipiras, E. , Gilbert‐Dussardier, B. , Benzacken, B. , Martin‐Coignard, D. , Henry, C. , David, A. , Lucas, J. , … Dubourg, C. (2010). Identification of gene copy number variations in patients with mental retardation using array‐CGH: Novel syndromes in a large French series. European Journal of Medical Genetics, 53(2), 66–75. 19878743 10.1016/j.ejmg.2009.10.002

[mgg31649-bib-0008] Kitsiou‐Tzeli, S. , Frysira, H. , Giannikou, K. , Syrmou, A. , Kosma, K. , Kakourou, G. , Leze, E. , Sofocleous, C. , Kanavakis, E. , & Tzetis, M. (2012). Microdeletion and microduplication 17q21.31 plus an additional CNV, in patients with intellectual disability, identified by array‐CGH. Gene, 492(1), 319–324. 22037486 10.1016/j.gene.2011.10.023

[mgg31649-bib-0009] Koolen, D. A. , Kramer, J. M. , Neveling, K. , Nillesen, W. M. , Moore‐Barton, H. L. , Elmslie, F. V. , Toutain, A. , Amiel, J. , Malan, V. , Tsai, A. C. , Cheung, S. W. , Gilissen, C. , Verwiel, E. T. , Martens, S. , Feuth, T. , Bongers, E. M. , de Vries, P. , Scheffer, H. , Vissers, L. E. … de Vries, B. B. (2012). Mutations in the chromatin modifier gene KANSL1 cause the 17q21.31 microdeletion syndrome. Nature Genetics, 44(6), 639–641. 22544363 10.1038/ng.2262

[mgg31649-bib-0025] Koolen, D. A. , Pfundt, R. , Linda K. , Beunders G. , Veenstra‐Knol H. E. , Conta J. H. , Fortuna A. M. , Gillessen‐Kaesbach G. , Dugan S. , Halbach S. , Abdul‐Rahman O. A. , Winesett H. M. , Chung W. K. , Dalton M. , Dimova P. S. , Mattina T. , Prescott K. , Zhang H. Z. , Saal H. M. , … de Vries B. B. A. (2016). The Koolen‐de Vries syndrome: A phenotypic comparison of patients with a 17q21.31 microdeletion versus a KANSL1 sequence variant. European Journal of Human Genetics, 24(5), 652–659. 10.1038/ejhg.2015.178 26306646 PMC4930086

[mgg31649-bib-0010] Koolen, D. A. , Sharp, A. J. , Hurst, J. A. , Firth, H. V. , Knight, S. J. L. , Goldenberg, A. , Saugier‐Veber, P. , Pfundt, R. , Vissers, L. E. L. M. , Destree, A. , Grisart, B. , Rooms, L. , Van der Aa, N. , Field, M. , Hackett, A. , Bell, K. , Nowaczyk, M. J. M. , Mancini, G. M. S. , Poddighe, P. J. , … de Vries, B. B. A. (2008). Clinical and molecular delineation of the 17q21.31 microdeletion syndrome. Journal of Medical Genetics, 45(11), 710–720. 18628315 10.1136/jmg.2008.058701 PMC3071570

[mgg31649-bib-0011] Koolen, D. A. , Vissers, L. E. , Pfundt, R. , de Leeuw, N. , Knight, S. J. , Regan, R. , Kooy, R. F. , Reyniers, E. , Romano, C. , Fichera, M. , Schinzel, A. , Baumer, A. , Anderlid, B. M. , Schoumans, J. , Knoers, N. V. , van Kessel, A. G. , Sistermans, E. A. , Veltman, J. A. , Brunner, H. G. , & de Vries, B. B. (2006). A new chromosome 17q21.31 microdeletion syndrome associated with a common inversion polymorphism. Nature Genetics, 38(9), 999–1001. 16906164 10.1038/ng1853

[mgg31649-bib-0012] Leonard, H. , & Wen, X. (2002). The epidemiology of mental retardation: challenges and opportunities in the new millennium. Mental Retardation and Developmental Disabilities Research Reviews, 8(3), 117–134. 12216056 10.1002/mrdd.10031

[mgg31649-bib-0013] Miller, D. T. , Adam, M. P. , Aradhya, S. , Biesecker, L. G. , Brothman, A. R. , Carter, N. P. , Church, D. M. , Crolla, J. A. , Eichler, E. E. , Epstein, C. J. , Faucett, W. A. , Feuk, L. , Friedman, J. M. , Hamosh, A. , Jackson, L. , Kaminsky, E. B. , Kok, K. , Krantz, I. D. , Kuhn, R. M. , … Ledbetter, D. H. (2010). Consensus statement: chromosomal microarray is a first‐tier clinical diagnostic test for individuals with developmental disabilities or congenital anomalies. American Journal of Human Genetics, 86(5), 749–764. 20466091 10.1016/j.ajhg.2010.04.006 PMC2869000

[mgg31649-bib-0014] Sharkey, F. H. , Morrison, N. , Murray, R. , Iremonger, J. , Stephen, J. , Maher, E. , Tolmie, J. , & Jackson, A. P. (2009). 17q21.31 microdeletion syndrome: further expanding the clinical phenotype. Cytogenetic and Genome Research, 127(1), 61–66. 20110647 10.1159/000279260

[mgg31649-bib-0015] Sharp, A. J. , Hansen, S. , Selzer, R. R. , Cheng, Z. , Regan, R. , Hurst, J. A. , Stewart, H. , Price, S. M. , Blair, E. , Hennekam, R. C. , Fitzpatrick, C. A. , Segraves, R. , Richmond, T. A. , Guiver, C. , Albertson, D. G. , Pinkel, D. , Eis, P. S. , Schwartz, S. , Knight, S. J. , & Eichler, E. E. (2006). Discovery of previously unidentified genomic disorders from the duplication architecture of the human genome. Nature Genetics, 38(9), 1038–1042. 16906162 10.1038/ng1862

[mgg31649-bib-0016] Shaw‐Smith, C. , Pittman, A. M. , Willatt, L. , Martin, H. , Rickman, L. , Gribble, S. , Curley, R. , Cumming, S. , Dunn, C. , Kalaitzopoulos, D. , Porter, K. , Prigmore, E. , Krepischi‐Santos, A. C. , Varela, M. C. , Koiffmann, C. P. , Lees, A. J. , Rosenberg, C. , Firth, H. V. , de Silva, R. , & Carter, N. P. (2006). Microdeletion encompassing MAPT at chromosome 17q21.3 is associated with developmental delay and learning disability. Nature Genetics, 38(9), 1032–1037. 16906163 10.1038/ng1858

[mgg31649-bib-0017] Smith, E. R. , Cayrou, C. , Huang, R. , Lane, W. S. , Cote, J. , & Lucchesi, J. C. (2005). A human protein complex homologous to the Drosophila MSL complex is responsible for the majority of histone H4 acetylation at lysine 16. Molecular and Cellular Biology, 25(21), 9175–9188. 16227571 10.1128/MCB.25.21.9175-9188.2005 PMC1265810

[mgg31649-bib-0018] Tan, T. Y. , Aftimos, S. , Worgan, L. , Susman, R. , Wilson, M. , Ghedia, S. , Kirk, E. P. , Love, D. , Ronan, A. , Darmanian, A. , Slavotinek, A. , Hogue, J. , Moeschler, J. B. , Ozmore, J. , Widmer, R. , Bruno, D. , Savarirayan, R. , & Peters, G. (2009). Phenotypic expansion and further characterisation of the 17q21.31 microdeletion syndrome. Journal of Medical Genetics, 46(7), 480–489. 19447831 10.1136/jmg.2008.065391

[mgg31649-bib-0019] Terrone, G. , D'Amico, A. , Imperati, F. , Carella, M. , Palumbo, O. , Gentile, M. , Canani, R. B. , Melis, D. , Romano, A. , Parente, I. , Riccitelli, M. , & Del Giudice, E. (2012). A further contribution to the delineation of the 17q21.31 microdeletion syndrome: central nervous involvement in two Italian patients. European Journal of Medical Genetics, 55(8–9), 466–471. 22659270 10.1016/j.ejmg.2012.04.010

[mgg31649-bib-0020] Tomlinson, M. W. , Treadwell, M. C. , & Bottoms, S. F. (1997). Isolated mild ventriculomegaly: associated karyotypic abnormalities and in utero observations. Journal of Maternal‐Fetal and Neonatal Medicine, 6(4), 241–244. 10.1002/(SICI)1520-6661(199707/08)6:4<241::AID-MFM11>3.0.CO;2-H 9260124

[mgg31649-bib-0021] Vallespín, E. , Palomares Bralo, M. , Mori, M. Á. , Martín, R. , García‐Miñaúr, S. , Fernández, L. , de Torres, M. L. , García‐Santiago, F. E. , Mansilla, E. , Santos, F. , M‐Montaño, V. E. , Crespo, M. C. , Martín, S. , Martínez‐Glez, V. , Delicado, A. , Lapunzina, P. , & Nevado, J. (2013). Customized high resolution CGH‐array for clinical diagnosis reveals additional genomic imbalances in previous well‐defined pathological samples. American Journal of Medical Genetics. Part A, 161A(8), 1950–1960. 23798500 10.1002/ajmg.a.35960

[mgg31649-bib-0022] Wray, C. D. (2013). 17q21.31 microdeletion associated with infantile spasms. European Journal of Medical Genetics, 56(1), 59–61. 23123321 10.1016/j.ejmg.2012.10.011

[mgg31649-bib-0023] Wright, E. B. , Donnai, D. , Johnson, D. , & Clayton‐Smith, J. (2011). Cutaneous features in 17q21.31 deletion syndrome: a differential diagnosis for cardio‐facio‐cutaneous syndrome. Clinical Dysmorphology, 20(1), 15–20. 21084979 10.1097/MCD.0b013e32833e8f1e PMC3000393

[mgg31649-bib-0024] Zollino, M. , Orteschi, D. , Murdolo, M. , Lattante, S. , Battaglia, D. , Stefanini, C. , Mercuri, E. , Chiurazzi, P. , Neri, G. , & Marangi, G. (2012). Mutations in KANSL1 cause the 17q21.31 microdeletion syndrome phenotype. Nature Genetics, 44(6), 636–638. 22544367 10.1038/ng.2257

